# College and Hospital Notes

**Published:** 1908-08

**Authors:** 


### COLLEGE AND HOSPITAL NOTES.

THE College of Medicine, Syracuse University, announces the following faculty and curriculum changes: Frank P. Knowlton, A. M., M. D., associate professor of physiology, has been made Professor of Physiology. H. S. Steensland, B. S., M. D., associate professor of pathology and bacteriology, has been made Professor of Pathology and Bacteriology. H. D. Senior, M. B., F. R. C. S., associate professor of anatomy, has been made Professor of Anatomy. Ernest N. Pattee, M. S., professor of chemistry in the College of Liberal Arts, has been made a member of the faculty of the College of Medicine. Richard H. Hutchings, M. D., Medical Superintendent Saint Lawrence State Hospital, Ogdensburg, N. Y., has been appointed Lecturer on Psychiatry. Ralph R. Fitch, M. D., of Rochester, N. Y. has been appointed Lecturer on Orthopedics. Charles V. Morrill, A. M., recently assistant in Zoölogy in Columbia University, New York, has been appointed Lecturer on Histology and Embryology.

Commencing in 1909, students entering the College of Medicine must have satisfactorily completed one full year, and on and after October, 1910, two full years in a science or arts course in a college recognised by the regents of the State of New York, and in that course and in their preparation for it a competent course in physics, chemistry, Latin, one modern language and biology must be included. The equivalent of this requirement, that is, evidence of having passed college examinations for admission to the sophomore or junior class is a recognised college by a student possessed of a medical student certificate from the State Educational Department, will be accepted. Hereafter all chemistry except applied chemistry will be taught in the new Bowne chemical laboratory of the College of Liberal Arts instead of in the College of Medicine as heretofore.

